# Optogenetic elevation of endogenous glucocorticoid level in larval zebrafish

**DOI:** 10.3389/fncir.2013.00082

**Published:** 2013-05-06

**Authors:** Rodrigo J. De Marco, Antonia H. Groneberg, Chen-Min Yeh, Luis A. Castillo Ramírez, Soojin Ryu

**Affiliations:** Developmental Genetics of the Nervous System, Max Planck Institute for Medical ResearchHeidelberg, Germany

**Keywords:** glucocorticoids, stress response, HPA axis, optogenetics, larval zebrafish

## Abstract

The stress response is a suite of physiological and behavioral processes that help to maintain or reestablish homeostasis. Central to the stress response is the hypothalamic-pituitary-adrenal (HPA) axis, as it releases crucial hormones in response to stress. Glucocorticoids (GCs) are the final effector hormones of the HPA axis, and exert a variety of actions under both basal and stress conditions. Despite their far-reaching importance for health, specific GC effects have been difficult to pin-down due to a lack of methods for selectively manipulating endogenous GC levels. Hence, in order to study stress-induced GC effects, we developed a novel optogenetic approach to selectively manipulate the rise of GCs triggered by stress. Using this approach, we could induce both transient hypercortisolic states and persistent forms of hypercortisolaemia in freely behaving larval zebrafish. Our results also established that transient hypercortisolism leads to enhanced locomotion shortly after stressor exposure. Altogether, we present a highly specific method for manipulating the gain of the stress axis with high temporal accuracy, altering endocrine and behavioral responses to stress as well as basal GC levels. Our study offers a powerful tool for the analysis of rapid (non-genomic) and delayed (genomic) GC effects on brain function and behavior, feedbacks within the stress axis and developmental programming by GCs.

## Introduction

Living organisms respond to stress by activating a complex repertoire of tightly regulated processes. These processes aim to preserve homeostasis and are collectively referred to as the stress response (Selye, 1956; Chrousos, 1998). Evolving adaptive responses to stress is essential for survival. However, dysfunctional stress responses can have devastating consequences for health and have been associated with a number of disorders, ranging from heart and vascular problems to depression, schizophrenia, and affective disorders (Holsboer et al., 1984; Nemeroff et al., 1984; Raber, 1998; De Kloet et al., 2005; McEwen, 2008; Yehuda, 2009). Despite their significance, the development of appropriate stress responsiveness and the mechanisms underlying stress response dysfunction remain largely unknown.

The stress response is mediated by the sympathetic nervous system and the hypothalamic-pituitary-adrenal (HPA) axis. While the sympathetic nervous system is responsible for the so called immediate “fight-or-flight” reactions, the HPA axis regulates both rapid and long-term stress effects (Charmandari et al., 2005). At the core of the HPA axis are corticotropin-releasing-hormone (CRH) and arginine vasopressin (AVP)-expressing neurons in the paraventricular nucleus (PVN) of the rostral hypothalamus, which respond to stress by triggering the release of adrenocorticotrophin hormone (ACTH) from the anterior pituitary. ACTH then stimulates glucocorticoid secretion from the adrenal glands. Glucocorticoids (GCs) are thus the final effectors of the HPA axis, with numerous targets both in the central nervous system and the periphery (Sapolsky et al., 2000).

GCs are known to influence brain function through genomic mechanisms via binding to two ligand-driven transcription factors, the high affinity mineralocorticoid receptor (MR) and the low affinity glucocorticoid receptor (GR), which contribute to delayed GC effects by regulating gene expression (De Kloet et al., 1998). Non-genomic GC effects on neuronal responses and behavior have also been reported, although the mechanisms underlying these rapid effects remain largely unknown (Dallman, 2005; Evanson et al., 2010; Groeneweg et al., 2011). It also remains unknown how non-genomic and genomic GC actions interact with each other to coordinate the activation and inhibition of different processes in multiple brain areas.

The analysis of GC effects under stress, particularly the rapid, non-genomic GC actions, has been hampered by the fact that GC release is tightly couple to that of other hormones. Also, GCs regulate vital functions under non-stress conditions, such as cell proliferation (Dickmeis and Foulkes, 2011). Therefore, elevating GC level is not sufficient to address the role of GCs under stress. It becomes necessary to specifically alter GCs levels under stressful situations triggered by stimuli of known intensity and endocrine effects. Current methods for altering GC levels entail either exposure to stressors or infusions of exogenous GCs. These methods are limited, however. Stressor exposure fails to selectively alter the rate of GC change, as it increases GC levels only via the stimulation of other stress hormones. GC infusion is not straightforward and can be stressful in itself, making it difficult to assess the impact of the treatment. To advance the analysis of stress correlates in the brain, it is paramount to examine GC actions as a function of time with increased specificity and temporal accuracy.

Because the stress response is conserved across phyla, zebrafish, *Danio rerio*, can aid in dissecting the complexity of GC actions. The zebrafish hypothalamic-pituitary-interrenal (HPI) axis shares key similarities with the HPA axis, with cortisol being the main circulating glucocorticoid in both humans and teleosts (Wendelaar Bonga, 1997; Flik et al., 2006). The preoptic nucleus in teleosts is considered a structure homologous to the mammalian PVN (Peter, 1977; Forlano and Cone, 2007). Adult zebrafish show increased cortisol levels and behavioral stress reactions upon stressor exposure (Ramsay et al., 2006, 2009; Speedie and Gerlai, 2008; Egan et al., 2009; Cachat et al., 2010; Steenbergen et al., 2011). Larval zebrafish also respond to stressors with increased cortisol levels (Alsop and Vijayan, 2008; Alderman and Bernier, 2009; Fuzzen et al., 2010; Clark et al., 2011; Steenbergen et al., 2011). Further, both their basal cortisol levels and expression levels of genes involved in corticosteroid synthesis and signaling increase drastically around the time of hatching, uncovering a stress response system that matures early in development (Alsop and Vijayan, 2008; Alderman and Bernier, 2009). Also importantly, interactions of GRs and serotonin signaling are conserved in zebrafish (Griffiths et al., 2012; Ziv et al., 2012).

In this report, we present a novel protocol for studying stress-induced GC effects. We used larval zebrafish to develop an optogenetic approach aimed at increasing the gain of the stress axis, so as to achieve different levels of endogenous GCs in response to a similarly stressful event. To this end, we expressed photoactivated adenylyl cyclase (bPAC) (Ryu et al., 2010; Stierl et al., 2011) specifically in pituitary cells, which govern GC secretion via ACTH release. Using light as a stressor as well as a source of stimulation for optogenetic control, we could induce both transient and persistent states of hypercortisolaemia in a highly controlled fashion. Importantly, optogenetically elevated GCs also enhanced locomotion shortly after stressor exposure, in line with the fact that stress mobilizes energy via GC signaling (Sapolsky et al., 2000). Our work established a powerful approach to modify the gain of the stress axis, making it possible to examine stress-dependent GC effects with high specificity and temporal accuracy. It provides a valuable tool for the analysis of rapid and delayed GC actions, interactions within the stress axis and feedbacks regulating endocrine and behavioral responses to stress.

## Materials and methods

### Generation of transgenic zebrafish

cDNA encoding myc-tagged bPAC from the soil bacterium *Beggiatoa* bPAC (Stierl et al., 2011) was PCR amplified with a mutated stop-codon and cloned into a vector containing a viral 2A sequence (Tang et al., 2009) and a fluorescent tdTomato marker flanked by I-SceI and Tol2 transposon recognition sites in the pBR322 backbone. This construct was combined with a fragment of the Pomc promoter, which was PCR amplified from a Pomc-GFP construct (Liu et al., 2003). The Pomc:bPAC-2A-tdTomato plasmid was incubated with 100 ng Tol2 transposase RNA for 10 min and injected in the presence of 0.05% phenol red into wild-type embryos (cross of AB and TL strains) in the one-cell stage. For further propagation of the transgenic line, we selected one founder, *Tg(Pomc:bPAC-2A-tdTomato)hd10*, with specific tdTomato expression in the pituitary and no ectopic expression.

### Zebrafish husbandry

Zebrafish breeding and maintenance was performed under standard conditions (Westerfield, 2000). Embryos were collected in the morning and raised on a 12:12 light/dark cycle in E2 medium (Westerfield, 2000). *Tg(Pomc:bPAC-2A-tdTomato)hd10* were crossed with wild-type fish and their progenies selected for the presence of tdTomato expression in the pituitary at 4 or 5 days post fertilization (dpf) using a fluorescent dissecting microscope. To avoid unspecific activation of bPAC prior to the experiments, transgenic embryos were raised in custom-made reflective containers covered by 550 nm long-pass filters (Thorlabs). Zebrafish experimental procedures were performed according to the guidelines of the German animal welfare law and approved by the local government.

### cAMP measure

50 pg capped *bPAC* RNA was prepared using a commercial mRNA kit (mMessage T7 Ultra Kit, Ambion) and injected into one-cell-stage wild-type embryos. Embryos were maintained under filtered light (see above) and subjected to blue-light stimulation at 1 dpf using the stimulation protocol described below (light power: 2.8 mW^*^cm^−2^). Groups of 27 embryos were collected immediately after the light-offset and homogenized in 0.1 M HCl on ice. After centrifugation, the supernatant was stored at −20°C. cAMP level was measured following the acetylation protocol from a cAMP ELISA kit (Enzo Life Sciences). Samples from light-stimulated *bPAC*-injected embryos were diluted 15 times in order to obtain values within the standard range.

### Immunohistochemistry

6 dpf larvae were fixed overnight at 4°C in 4% paraformaldehyde (PFA) in phosphate-buffered saline (PBS). Immunohistochemistry was performed as previously described (Ryu et al., 2007), using either polyclonal antibody against human ACTH (National Hormone and Peptide Program, National Institute of Diabetes and Digestive and Kidney Diseases, 1:500) or rabbit polyclonal antibody against Myc-Tag (Cell Signaling Technology, 1:500) as primary antibodies, and Alexa Fluor 488 anti-rabbit (Invitrogen, 1:1000) as a secondary antibody. Detection of residual tdTomato fluorescence after the fixation did not require immunohistochemistry. Larvae were imaged in 80% glycerol using a Nikon 20x glycerol objective and a Leica SP5 CLSM. Confocal image stacks were subsequently evaluated using Amira 5.4 (Visualization Sciences Group) to create maximum intensity projections.

### Cortisol ELISA

For cortisol detection, groups of 30 larvae (6 dpf) were immobilized in ice water, frozen in ethanol/dry ice bath, and stored at −20°C. Cortisol from homogenized samples was extracted with ethyl acetate. We employed a home-made cortisol ELISA protocol (C. M. Yeh, M. Glöck, R. J. De Marco, S. Ryu, unpublished data), using cortisol mouse antibody (EastCoast Bio), cortisol standards (Hydrocortisone, Sigma-Aldrich) and cortisol-HRP (EastCoast Bio). The reactions were stopped using 1M sulfuric acid and read at 450 nm in an ELISA reader (Multiskan Ascent, Thermo Scientific). The data were corrected for dilution factor, extraction efficiency, and recovery function. In all experiments, cortisol samples were taken 2 min after the offset of light, unless otherwise stated.

### Mifepristone incubation

6 dpf larvae were incubated for 2 h in 1 μM Mifepristone (RU-486, Sigma-Aldrich) dissolved in E2-Medium with 0.1% DMSO. This concentration has been shown to abolish a genomic GC response signal (Weger et al., 2012). During light stimulation, larvae were maintained in the Mifepristone solution to avoid further handling.

### Light stimulation

A custom-made LED ring was placed at a fixed distance above a mutiwell plate (for behavioral testing) or a single container (for cortisol extraction). The incident angle of the LEDs allowed for homogeneous illumination of the samples. We used custom-made drivers, pulse generators and a TTL control box (USB-IO box, Noldus) to control the LEDs. Larvae were exposed for 18 s or 180 s to either blue- or yellow-light of varying power, using single or multiple stimulation protocols. Each light pulse consisted of 100 ms flashes delivered at 5 Hz. Light power was measured using a hand-held light power meter (Newport). For the multiple stimulation protocol, we used three light pulses delivered with an inter-trial interval of 30 min.

### Early light stimulation

To facilitate light stimulation with a higher throughput, we arranged LEDs so as to homogeneously illuminate a six well plate with a light power of 0.6 mW^*^cm^−2^. At 4 dpf, we exposed the bPAC-positive (bPAC^+^) larvae and their negative siblings (bPAC^−^) to the above described multiple stimulation protocol. Next, the larvae were placed back in the incubator and kept in E2 medium inside the reflective containers covered by the 550 nm long-pass filters. We repeated this procedure 24 h later. At the end of 5 dpf, we screened the larvae for tdTomato expression in the pituitary. At 6 dpf, both the bPAC^+^ and bPAC^−^ larvae subjected to the above protocol were either directly collected for measuring basal cortisol levels or first stimulated with a single 180 s squared pulse of blue-light (0.6 mW^*^cm^−2^) and then collected for measuring light-induced cortisol change. Control animals for each group were handled in the same fashion, but omitting the light stimulation at 4 and 5 dpf. Stimulations were performed within a fixed 3 h window during the larvae's day time.

### Behavioral testing

Behavioral tests were performed using wild-type, bPAC^+^ and bPAC^−^ 6 dpf larvae. Experiments were conducted under infrared (IR) light, delivered through an array of IR-LEDs mounted inside a custom-made light-proof enclosure placed on a vibration-free platform (Newport). We used an infrared-sensitive camera (ICD-49E B/W, Ikegami Tsushinki) to image the movements of the swimming larvae at 25 frames s^−1^. The lens of the camera (TV Lens, Computer VARI FOCAL H3Z4512 CS-IR, CBC) was surrounded by a custom-made LED ring and positioned above a multiwell plate (Greiner-Bio One). We used EthoVision XT software (Noldus Information Technology) to simultaneously track the movements of 30 larvae swimming individually inside the wells in 50 μL of E2 medium. In all experiments, the larvae were allowed to adjust to the test conditions for 15 min prior to the recordings. Experiments were conducted at room temperature. We continuously monitored the temperature inside a reference well using a thermocouple (npi electronics) connected to a temperature control system (PTC 20, npi electronics; Exos-2 V2 liquid cooling system, Koolance). All the experiments were performed in a blind fashion using unscreened larvae to avoid effects of pre-handling and exposure to unfiltered light. Tests were conducted between 9:00 and 18:00 and the different experimental groups intermixed throughout the day.

### Statistical analysis

All data are shown as mean and standard error of the mean (S.E.M.). To facilitate comparison, locomotor activity is expressed either as distance swam per unit of time (Figures 2A–C) or as percentual motion relative to pre-stimulation baseline levels (Figures 5A–C), as these levels did not differ between bPAC^+^ and bPAC^−^ larvae (Mann–Whitney test, *p* = 0.11). We used Student's *t*-tests (two-tailed) for two-group comparisons, or Mann–Whitney *U*-tests if the data did not fulfill the assumptions of the *t*-test. ANOVAs were used for multiple group comparisons, followed by Bonferroni's *post-hoc* tests, or their non-parametric equivalents. We also used repeated-measures linear regression analysis (Fitzmaurice et al., 2004). Analyzes were carried out using MS-Excel, Matlab 2009b (MathWorks), Prism 5, (Graphpad Software), Sigma Plot (Systat), R and Virtual Dub (Freeware).

## Results

### A brief light exposure is stressful for dark-adapted larvae

**Figure 1:**
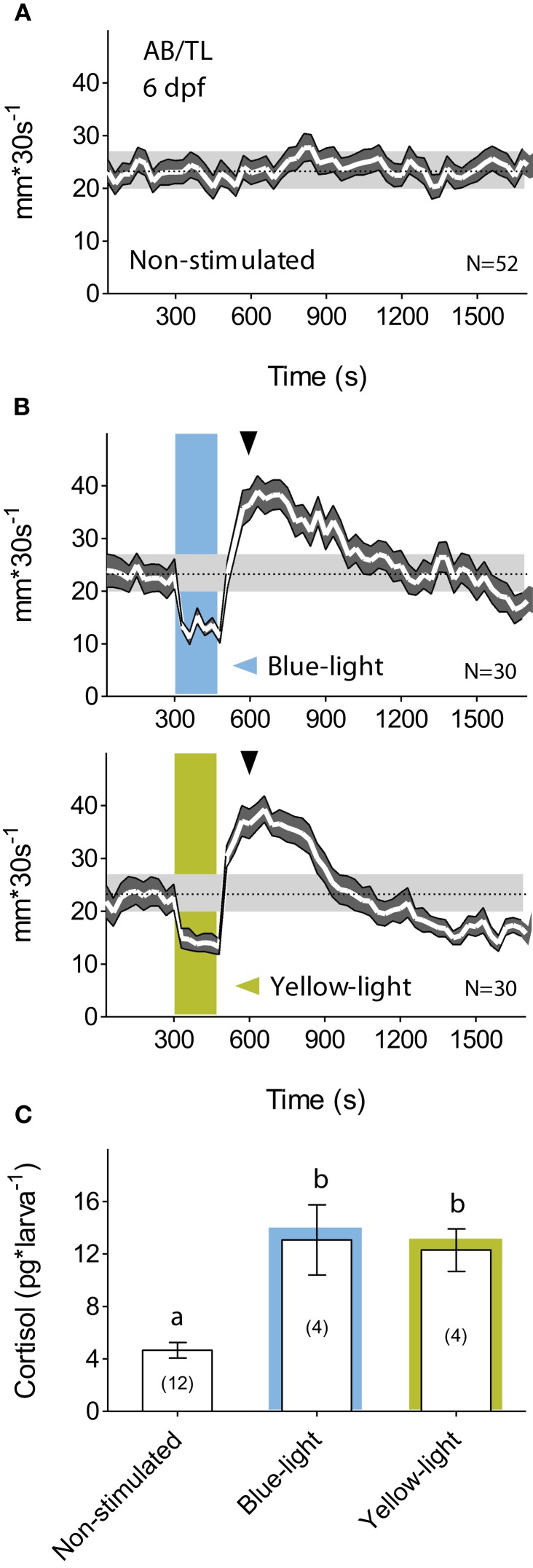
**A brief exposure to light is stressful for dark-adapted larvae. (A)** Wild-type 6 dpf larval zebrafish display regular motion levels while swimming in darkness (overall mean ± S.E.M. shown as dotted line and gray background, respectively). **(B)** When dark-adapted for 15 min, 6 dpf larvae react to a 180 s squared pulse of either blue- (top) or yellow-light (bottom) with reduced locomotion after the light-onset followed by increased locomotion after the light-offset. Afterward, locomotion decreases gradually until it reaches steady-state levels tens of minutes later (light-power: 2.8 mW^*^cm^−2^; gray arrowheads indicate cortisol extraction times). **(C)** Such a brief exposure to either blue- or yellow-light increases whole-body cortisol level (lowercase letters indicate statistical differences among groups; sample size in parenthesis).

The small transparent bodies of larval zebrafish make them suitable for non-invasive manipulation of neuronal activity using light. Yet, larval zebrafish are highly sensitive to photic stimuli (Burgess and Granato, 2007). While swimming in darkness, for example, they display stable rates of discontinuous motion (Figure 1A) and react to a brief exposure to light with stereotyped changes in locomotor activity (Macphail et al., 2009). First, they show a drastic reduction of locomotion after the light onset, followed by increased locomotion after the light-offset. Afterward, locomotion decreases gradually until it reaches steady-state levels several minutes later. A drastic reduction of locomotion in response to external stimulation is generally thought of as a fear-related response. Many species secrete cortisol in threatening situations associated with greater fear. Hence, as a prerequisite to develop optogenetic approaches for stress research, we set up to examine the effect of illumination change on locomotion and cortisol level. Since optogenetic photo-actuators work upon absorption of a wide range of light wavelengths, we first tested whether the larval stereotyped reactions to illumination change could be similarly evoked by a squared pulse of either blue- or yellow-light. We observed that both light wavelengths elicited similar motion patterns (Figure 1B). Next, we examined the extent to which such a brief exposure to light could act as a stressful event, and observed that 6 dpf larvae reacted to a 180 s squared pulse of either blue- or yellow-light with increased cortisol levels (Figure 1C; Mann–Whitney test, blue-light: *p* < 0.01, yellow-light: *p* = 0.03), thereby specifying that a squared pulse of light can act as a stressful input signal. This effect of a fast transition from darkness to light could not be accounted for by “wakefulness” variations as reflected in motion. Larvae kept for such a brief period of time either under constant white illumination or in complete darkness displayed similar levels of locomotor activity [*t*-test, *t*_(24)_ = 0.4, *p* = 0.72].

### Increasing the gain of the stress axis

**Figure 2:**
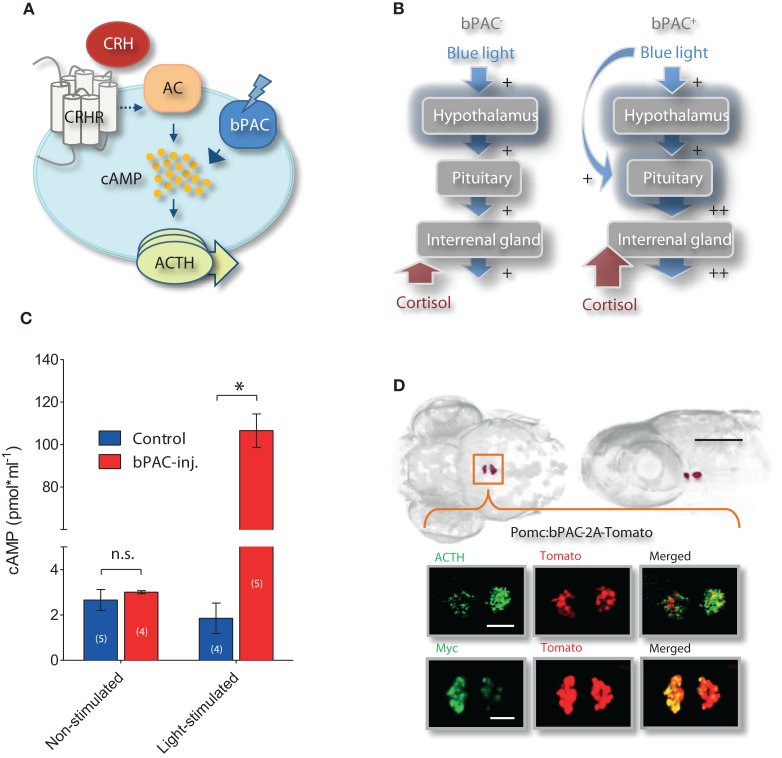
**Optogenetic increase of the gain of the stress axis. (A)** In pituitary corticotrophs, *Beggiatoa* photoactivated adenylyl cyclase (bPAC) is expected to amplify CRH signaling and ACTH release; CRHR, CRH receptor; AC, adenylyl cyclase. **(B)** We aimed to modify the gain of the HPI axis by targeting bPAC to pituitary corticotrophs. Based on this rationale, blue-light stimulation of bPAC is expected to enhance the increase in cAMP that is central to CRH signaling in corticotroph cells, thereby amplifying ACTH and subsequent cortisol release while preserving analogous levels of hypothalamus activation. According to this scheme, stress-induced over-elevation of cortisol would be varied by modifying the light-power and/or duration of the squared pulse of blue-light. **(C)** Blue-light dependent rise in whole-body cAMP level in 1 dpf larvae using bPAC RNA (asterisks indicate statistical difference between groups at *p* < 0.05). **(D)** Dorsal and lateral views of bPAC expression in two cell clusters in the pituitary of 6 day post fertilization (dpf) larvae (scale bar: 500 μm), as detected by fused tdTomato fluorescence; co-expression of ACTH and fluorescent tdTomato signal (top), and of myc-tag and tdTomato signal (bottom); scale bars: 50 μm.

Since light in itself can stimulate stress networks, we reasoned that the presence of photo-actuators within the HPI axis would allow us to meaningfully alter its light-triggered activation. In particular, we aimed to manipulate the increase of cortisol triggered by light so as to induce greater and controllable rates of cortisol rise in response to otherwise similarly stressful events. Technically speaking, this means that we aimed to increase the gain of the stress axis by amplifying the output (cortisol) of a constant input signal (light). To this end, we chose to target the expression of *Beggiatoa* bPAC (Ryu et al., 2010; Stierl et al., 2011) specifically to ACTH-producing pituitary corticotroph cells. Stress activates complex intracellular CRH signaling cascades in multiple cell types (Arzt and Holsboer, 2006). In pituitary cells, an increase in cAMP downstream of CRH receptor activation causes ACTH release. We therefore hypothesized that blue-light stimulation of bPAC will lead to increased cAMP levels in pituitary corticotrophs and, consequently, also to enhanced ACTH release (Figure 2A). Enhanced levels of circulating ACTH will then be expected to co-vary with whole-body cortisol (Figure 2B), as the melanocortin receptor type 2 (MC2R) is predominantly expressed in the interrenal gland and not in the zebrafish brain (Agulleiro et al., 2010). We first demonstrated that bPAC is functional in zebrafish larvae. Injecting *bPAC* mRNA into embryos in the one-cell stage led to a blue-light dependent elevation of whole-body cAMP at 1 dpf (Figure 2C; Mann–Whitney test, *p* = 0.19 for non-stimulated control vs. bPAC-injected; *p* = 0.02 for light-stimulated control vs. *bPAC*-injected). To target bPAC specifically to pituitary corticotrophs, we used a fragment of the proopiomelanocortin (POMC) promoter whose expression pattern is restricted to corticotroph cells (Liu et al., 2003). To aid in visualization of the expression of the construct, the bPAC protein sequence was fused to myc-tag and a fluorescent reporter, tdTomato, via the viral 2A peptide. The transgenic line expressed bPAC specifically in pituitary corticotrophs, as revealed by the co-localization of ACTH with myc and tdTomato signal (Figure 2D).

### Optogenetic elevation of stress-induced cortisol level

**Figure 3:**
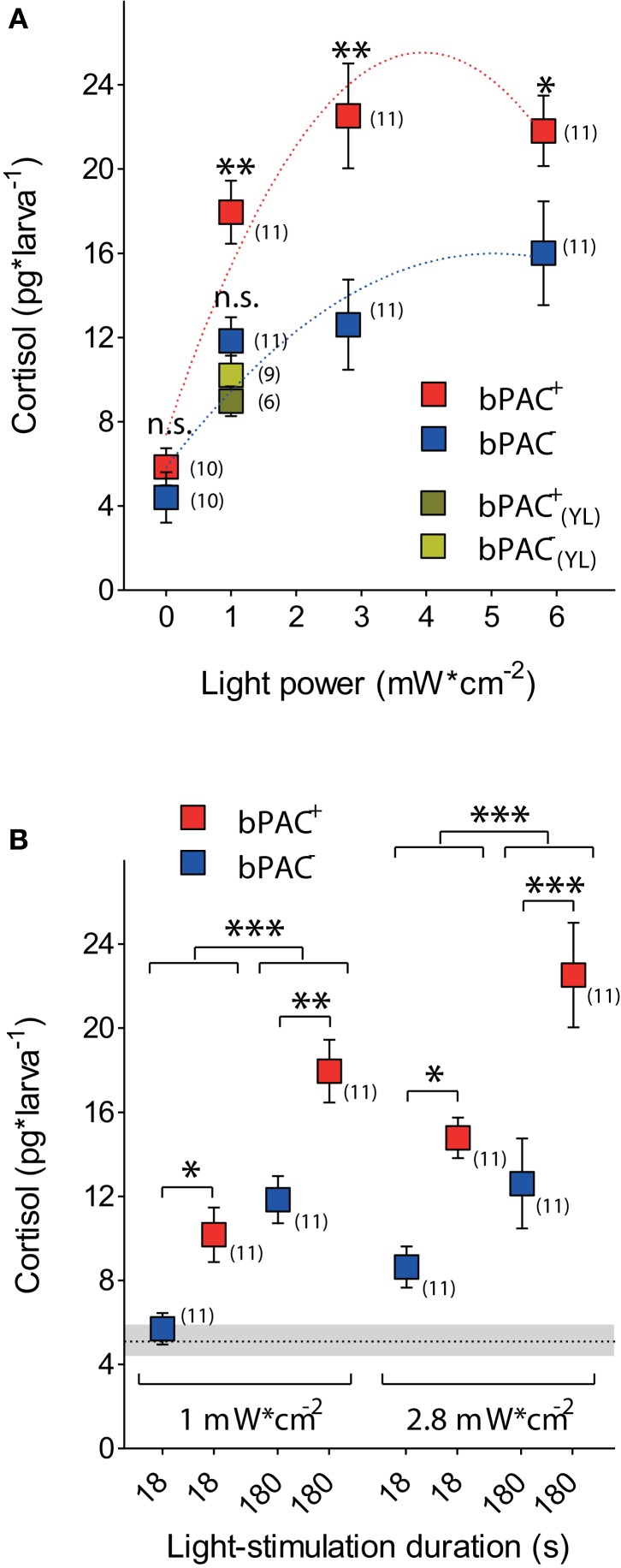
**Optogenetic elevation of stress-induced cortisol level. (A)** A 180 s squared pulse of blue-light leads to higher cortisol levels in bPAC-positive larvae (bPAC^+^) as compared to their negative siblings (bPAC^−^) (asterisks indicate statistical differences between groups at *p* < 0.05 or *p* < 0.01; sample size in parenthesis; the red and blue dashed lines depict significant non-linear regressions of cortisol vs. light-power for bPAC^+^ and bPAC^−^larvae, respectively). Note that yellow-light fails to differentially enhance cortisol level in bPAC^+^ larvae. **(B)** Cortisol level in bPAC^+^ and bPAC^−^ larvae as a function of exposure time and light-power (asterisks indicate statistical differences between groups at *p* < 0.05, *p* < 0.01, or *p* < 0.001; sample size in parenthesis; Mean ± S.E.M. basal levels shown as dotted line and gray background, respectively).

Consistent with our observations in wild-type larvae (Figures 1A–C), a 180 s squared pulse of blue-light led to increased cortisol levels in both bPAC-positive (bPAC^+^) and bPAC-negative (bPAC^−^) larvae. However, the former showed substantially higher cortisol levels (Figure 3A; Two-Way ANOVA, light power: *F*_(3, 82)_ = 29.48, *p* < 0.0001; genotype: *F*_(1, 82)_ = 23.09, *p* < 0.0001; light power X genotype: *F*_(3, 82)_ = 1.77, *p* = 0.16; followed by Bonferroni post-tests for within light-power pair comparisons). Yellow-light failed to enhance the rise of cortisol in the bPAC^+^ larvae (Figure 3A; One-Way ANOVA, *F*_(3, 36)_ = 10.73, *p* < 0.0001; followed by Bonferroni post-tests for bPAC^+^_blue_ vs. bPAC^−^_blue_, bPAC^+^_yellow_ or bPAC^−^_yellow_, and for bPAC^+^_yellow_ vs. either bPAC^−^_blue_ or bPAC^−^_yellow_), in line with the fact that bPAC activation is blue-light specific due to its BLUF (blue-light receptor using FAD) type light-sensor domain (Ryu et al., 2010; Stierl et al., 2011). Further, already the lowest light-power caused maximum differences between the cortisol levels of the bPAC^+^ and bPAC^−^ larvae (Figure 3A). This latter result led us to examine the effects of a shorter light stimulation. We then observed that the bPAC^+^ larvae showed enhanced cortisol levels in response to a ten times shorter stimulation, i.e., a light pulse lasting less than 20 s (Figure 3B; Two-Way ANOVA, left, length: *F*_(1, 40)_ = 33.85, *p* < 0.0001; genotype: *F*_(1, 40)_ = 19.56, *p* < 0.0001; length X genotype: *F*_(1, 40)_ = 0.47, *p* = 0.50; right, length: *F*_(1, 40)_ = 10.85, *p* = 0.002; genotype: *F*_(1, 40)_ = 20.37, *p* < 0.0001; length X genotype: *F*_(1, 40)_ = 1.13, *p* = 0.29; followed by Bonferroni post-test for pair comparisons), demonstrating that our approach allows for GC alterations with high temporal resolution.

### Multiple light stimulations lead to transient hypercortisolic states in bPAC^+^ larvae

**Figure 4:**
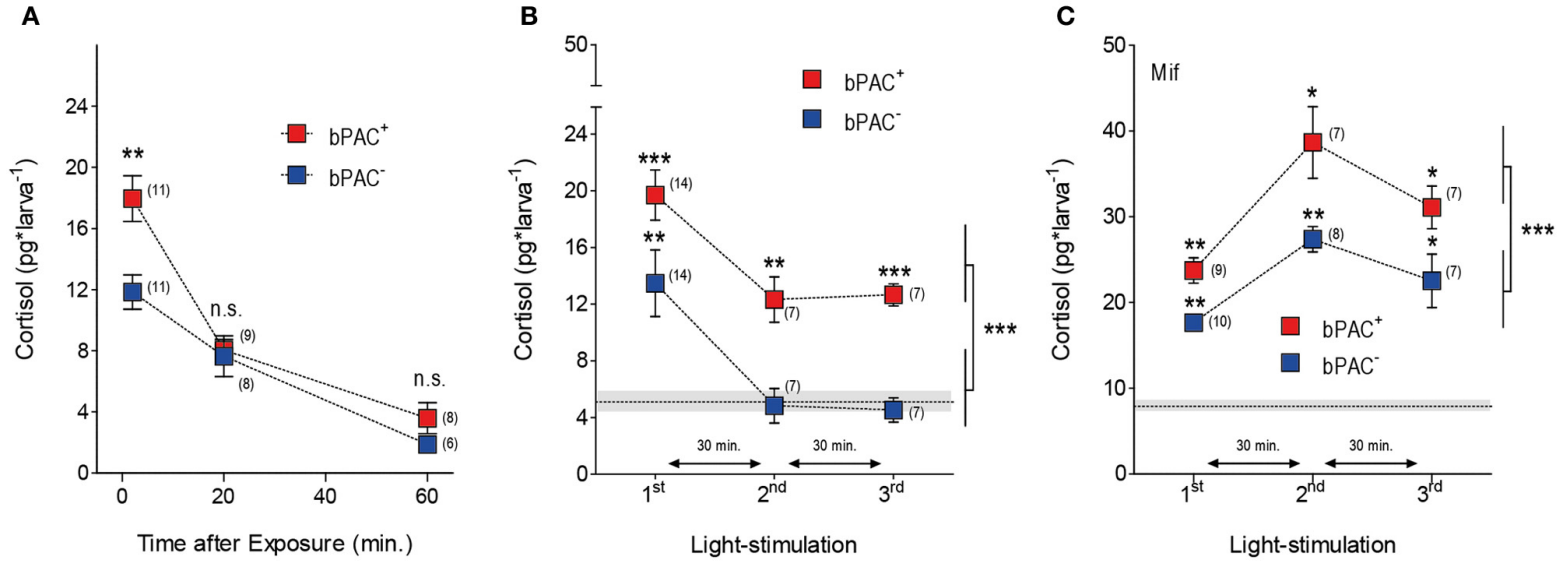
**Multiple light stimulations lead to hypercortisolic states in bPAC^+^ larvae. (A)** Light-induced cortisol level decreases as a function of time in both bPAC^+^ and bPAC^−^ larvae (asterisks indicate statistical diff-erences between groups at *p* < 0.001; light-power: 1 mW^*^cm^−2^, exposure time: 180 s). **(B)** bPAC^+^ but not bPAC^−^ larvae respond to a sequence of three 180 s squared pulses of blue-light with increased cortisol levels (asterisks indicate statistical differences between groups at *p* < 0.01 or *p* < 0.001; light-power: 2.8 mW^*^cm^−2^; inter-trial interval: 30 min). **(C)** In the presence of the GR antagonist mifepristone (Mif), both bPAC^+^ and bPAC^−^ larvae respond to multiple light stimulations with increased cortisol levels, which are, on average, substantially higher than those from non-incubated larvae (asterisks indicate statistical differences between groups at *p* < 0.05 or *p* < 0.01; light-power: 2.8 mW^*^cm^−2^; inter-trial interval: 30 min). **(B,C)** Mean basal cortisol level ± S.E.M. shown as a dotted line and gray background, respectively; note that basal cortisol levels are comparatively higher in the Mif-incubated larvae.

GCs regulate their own production by decreasing CRH and ACTH outputs from the hypothalamus and pituitary, respectively (Dallman and Yates, 1969; Dallman et al., 1994). We then asked whether optogenetic elevation of endogenous GCs could lead to transient hypercortisolic states repeatedly. After the differential rise of cortisol triggered by blue-light, both the bPAC^+^ and bPAC^−^ larvae had similar and significantly reduced cortisol levels 20 min after the light offset (Figure 4A; bPAC^+^, *F*_(2, 27)_= 38.74, *p* < 0.0001; bPAC^−^, *F*_(2, 24)_ = 17.70, *p* < 0.0001; *t*-test for pair comparisons within time points), indicating that cortisol-mediated negative feedback is fully functional in zebrafish larvae. Next, we repeatedly exposed groups of bPAC^+^ and bPAC^−^ larvae to a sequence of three 180 s squared pulses of blue-light. In order to compare cortisol values resulting from the most recent light pulse and not from previously elevated levels, we used a time interval of 30 min in-between light pulses, which assured similarly low levels in both groups at the time of the second and third pulses (Figure 4A). Using this multiple light stimulation protocol, we observed that the bPAC^+^ larvae responded to each of the light pulses with increased cortisol levels, whereas the bPAC^−^ larvae failed to do so after the first pulse (Figure 4B; Two-Way ANOVA, repeated exposure: *F*_(2, 50)_ = 12.44, *p* < 0.0001; genotype: *F*_(1, 50)_ = 18.55, *p* < 0.0001; repeated exposure X genotype: *F*_(2, 50)_ = 0.13, *p* = 0.88; one sample *t*-tests for comparisons against basal level). These results demonstrated that multiple light stimulations can repeatedly lead to hypercortisolic states in bPAC^+^ larvae, even if the HPI axis has been down-regulated by previously elevated GC levels. To verify the role of the cortisol-mediated negative feedback in this phenomenon, we applied the same stimulation protocol to bPAC^+^ and bPAC^−^ larvae that had been incubated with mifepristone (Mif), an antagonist for the GC-receptor (GR) that is effective in larval zebrafish (Weger et al., 2012). Under these circumstances, both the bPAC^+^ and bPAC^−^ larvae responded to each of the several light pulses with increased cortisol levels. These stress-induced levels were much higher than those from the non-incubated larvae, verifying that our multiple stimulation protocol leads to down-regulated HPI axis activity. Yet, the bPAC^+^ larvae still showed substantially higher cortisol levels than the bPAC^−^ larvae (Figure 4C; Two-Way ANOVA, repeated exposure: *F*_(2, 41)_ = 16.30, *p* < 0.0001; genotype: *F*_(1, 41)_ = 21.88, *p* < 0.0001; repeated exposure X genotype: *F*_(2, 41)_ = 0.72, *p* = 0.49; one sample *t*-tests for comparisons against basal level). Also, the basal cortisol levels of both groups of larvae were higher in the Mif-incubated larvae as compared to the non-incubated larvae (Figures 4B,C; Mann–Whitney test, *p* = 0.004). Taken together, these results show that our approach can be used to induce hypercortisolic states repeatedly, making it possible to examine the effect of repeated GC over-exposure on stress axis development and function.

### Optogenetically elevated cortisol level leads to enhanced locomotion after stressor exposure

**Figure 5:**
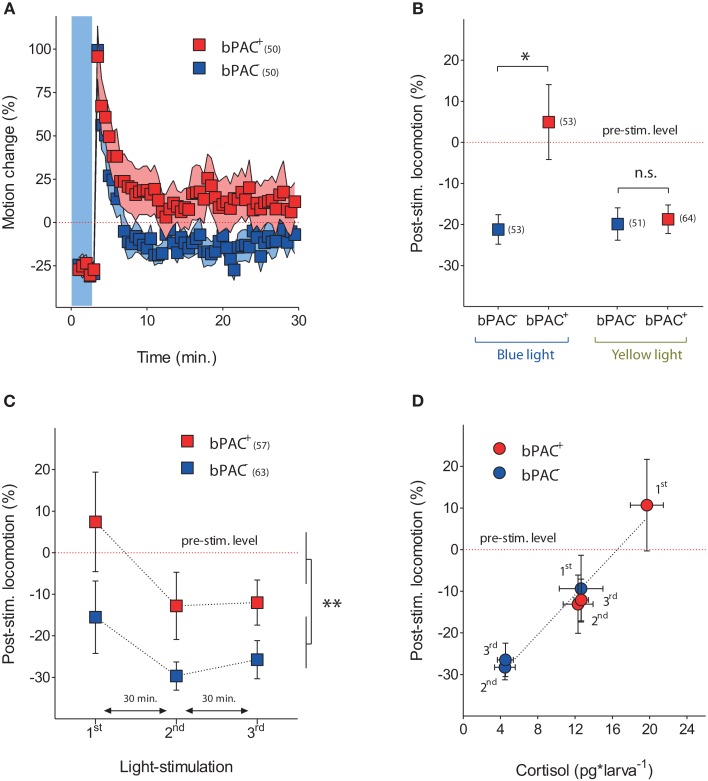
**Optogenetically elevated cortisol level leads to enhanced locomotion after stressor exposure. (A)** Locomotor activity in bPAC^+^ (red squares) and bPAC^−^ larvae (blue squares) during and after a 180 s squared pulse of blue-light (shown as blue background) (light-power: 2.8 mW^*^cm^−2^; sample size in parenthesis). **(B)** In bPAC^+^ larvae, a 180 s squared pulse of blue-light, but not of yellow-light, leads to enhanced locomotion (measured over a 10 min period) after the light offset. In bPAC^−^ larvae, by contrast, neither blue- nor yellow-light influences locomotion after the light-offset (asterisks indicate statistical difference between groups at *p* < 0.05; light-power: 1 mW^*^cm^−2^; sample size in parenthesis; see Materials and Methods for details on motion calculations). **(C)** Over multiple light exposures, post-stimulation locomotion is higher in the bPAC^+^ larvae than in the bPAC^−^ larvae (asterisks indicate statistical difference between the groups at *p* < 0.01; light-power: 2.8 mW^*^cm^−2^; sample size in parenthesis). **(D)** Locomotion levels from bPAC^+^ and bPAC^−^ larvae plotted against corresponding cortisol levels; note how post-stimulation locomotion shows linear dependence of past cortisol levels.

We noticed that blue-light led to higher post-stimulation locomotion in the bPAC^+^ larvae, as compared to their negative siblings (Figure 5A). Hence, we compared the steady-state post-stimulation motion levels of the bPAC^+^ and bPAC^−^ larvae 20 min after a single pulse of either blue- or yellow-light. We then observed that blue- but not yellow-light enhanced locomotion in the bPAC^+^ larvae, whereas neither blue- nor yellow-light enhanced locomotion in the bPAC^−^ larvae (Figure 5B; Mann–Whitney tests, blue-light: *p* < 0.04, yellow-light: *p* = 0.68). We also compared the post-stimulation motion levels of both groups using data from the multiple light stimulation protocol (Figure 4B). Once again, locomotion was higher in the bPAC^+^ than in the bPAC^−^ larvae (Figure 5C; Two-Way ANOVA, repeated exposure: *F*_(2, 306)_ = 3.0, *p* = 0.0513; genotype: *F*_(1, 306)_ = 8.26, *p* = 0.0043; repeated exposure X genotype: *F*_(2, 306)_ = 0.19, *p* = 0.83). Noticiably, the motion values from both groups of larvae plotted against the corresponding cortisol levels could be linearly approximated (Figure 5D; repeated-measures linear regression analysis, *p* < 0.001). These results indicated that a brief exposure to blue-light can cause not only hypercortisolic states in dark-adapted bPAC^+^ larvae, but also tightly correlated deviations from nominal locomotion.

### Early blue-light stimulation causes long-term hypercortisolaemia in bPAC^+^ larvae

**Figure 6:**
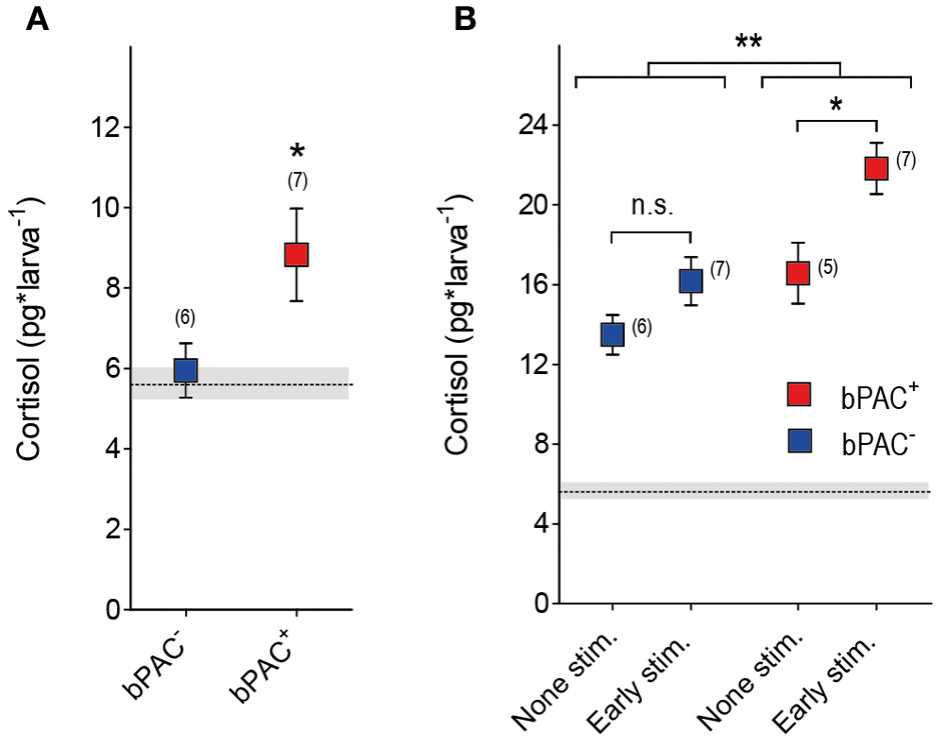
**Early blue-light stimulation causes long-term hypercortisolaemia in bPAC^+^ larvae. (A)** bPAC^+^ (red squares) but not bPAC^−^ larvae (blue squares) show increased basal cortisol levels after having being exposed to multiple light stimulations over 2 consecutive days (asterisks indicate statistical difference between groups at *p* < 0.05; light-power: 0.6 mW^*^cm^−2^; sample size in parenthesis). **(B)** At 6 dpf, bPAC^+^ larvae (red squares) exposed to light stimulation at 4 and 5 dpf (early stim.) respond to a squared pulse of blue-light with higher cortisol levels as compared to either none-exposed bPAC^+^ (non-stim.) or exposed and non-exposed bPAC^−^ larvae (asterisks indicate statistical differences between groups at *p* < 0.05 or *p* < 0.01; light-power: 0.6 mW^*^cm^−2^; sample size in parenthesis). **(A,B)** Mean basal cortisol level ± S.E.M. of both bPAC^+^ and bPAC^−^ larvae shown as a dotted line and gray background, respectively.

Early GC overexposure can lead to persistent alterations of HPA axis function (Kapoor et al., 2006; Seckl and Holmes, 2007; Seckl, 2008). We asked whether multiple light stimulations at early stages of development could lead to long-term forms of hypercortisolaemia in bPAC^+^ larvae. To answer this question, we applied the multiple light stimulation protocol (Figure 4) to the bPAC^+^ and bPAC^−^ larvae at 4 and 5 dpf. Later, at 6 dpf, we measured the basal and stress-induced cortisol levels of the larvae following a single 180 s squared pulse of blue-light (Figure 6). We then observed that the bPAC^+^ larvae had increased basal cortisol at 6 dpf (Figure 6A; Wilcoxon signed rank test, bPAC^+^: *p* = 0.03, bPAC^−^: *p* = 0.84), whereas the basal cortisol levels of the bPAC^+^ and bPAC^−^ larvae that had not been exposed to early blue-light stimulation did not differ from each other [*t*-test, *t*_(23)_ = 1.1, *p* = 0.31]. Also, the bPAC^+^ larvae responded to a light pulse with higher cortisol level, as compared to either the non-exposed bPAC^+^ larvae or the exposed and non-exposed bPAC^−^ larvae (Figure 6B; Two-Way ANOVA, early stimulation: *F*_(1, 21)_ = 9.8, *p* < 0.01; genotype: *F*_(1, 21)_ = 11.9, *p* < 0.01; early stimulation X genotype: *F*_(1, 21)_ = 1.0, *p* = 0.3, followed by Bonferroni post-tests for within genotype pair comparisons). These results demonstrated that early blue-light stimulation causes long-term hypercortisolaemia in bPAC^+^ larvae.

## Discussion

Here we provide evidence for optogenetic modification of the gain of stress axis in larval zebrafish. Expressing *Beggiatoa* bPAC (Ryu et al., 2010; Stierl et al., 2011) specifically in ACTH-producing pituitary corticotroph cells enhances the rise of endogenous cortisol triggered by stress. Using cell-specific optogenetic manipulation of cAMP levels *in vivo*, a home-made cortisol ELISA, and behavioral tracking, our experiments determined that blue-light can activate the stress axis and enhance the ensuing cortisol rise in bPAC^+^ larvae, also causing tightly correlated changes in locomotor activity. Additionally, our data demonstrated that early blue-light stimulation can lead to persistent forms of hypercortisolaemia in bPAC^+^ larvae. Altogether, we developed a tool suitable for the analysis of rapid and delayed effects of stress-associated glucocorticoid levels.

Our tests were specifically designed to amplify the activity of the stress axis non-invasively, maintaining cortisol levels within their physiological range. Upon absorption of blue-light, *bPAC* mRNA injected into embryos in the one-cell stage elevated whole-body cAMP at 1 dpf, verifying that bPAC is functional in zebrafish larvae (Figure 2C). The expression of bPAC was restricted to pituitary corticotroph cells, as we used a specific promoter and the fluorescence of the fused tdTomato marker was detected nowhere else in the transgenic embryo (Figure 2D). *Beggiatoa* PAC has the advantage of having a lower dark activity, as compared to previously reported versions of the enzyme (Schroder-Lang et al., 2007; Ryu et al., 2010; Stierl et al., 2011). Nevertheless, to prevent unspecific activation of bPAC by white light, transgenic embryos were raised under 550 nm long-pass filters. In line with this, both the basal cortisol levels and locomotion estimates of the bPAC^+^ larvae were similar to those of their negative siblings prior to the tests (Figure 3A). The blind design of the motion recordings prevented potential biases caused by any possible differential handling of the larvae. In addition, we randomly distributed groups and treatments throughout the day to avoid biased variability due to circadian cortisol variations (Dickmeis et al., 2007).

Stress causes glucocorticoid secretion via the coupled release of CRH and ACTH. Whereas ACTH primarily stimulates GC secretion, CRH and GCs have widely distributed receptors. Both CRH and GCs have been implicated in a variety of stress correlates, making it difficult to study their specific contributions to the stress response. GCs exert fast and delayed actions in multiple brain areas (Dallman, 2005; Evanson et al., 2010; Groeneweg et al., 2011). For instance, they act rapidly on neurons in the hippocampus (Komatsuzaki et al., 2005), amygdala (Karst et al., 2010), thalamus and caudate nucleus (Strelzyk et al., 2012), among other brain areas. GCs also feedback onto PVN neurons through genomic GR-mediated and non-genomic membrane-initiated mechanisms (Jones et al., 1976; De Kloet et al., 1998; Dallman, 2005; Malcher-Lopes et al., 2006; Di and Tasker, 2008; Evanson et al., 2010). Moreover, it has also been reported that inhibition of ACTH release from the anterior pituitary occurs via genomic as well as non-genomic GC actions (Jones et al., 1972; Kaneko and Hiroshige, 1978; Widmaier, 1984). In order to specify mechanisms underlying rapid and delayed GC effects under stress, it is necessary to control the rate of variation of endogenous GCs without modifying the activity of upstream hypothalamic networks. However, to date no effective method has been available to selectively modify the rate at which endogenous GC levels vary in response to stress.

Our experiments established a 270% increase of whole-body cortisol level within the first 5 min after the onset of a 180 s squared pulse of either blue- or yellow-light in dark-adapted wild-type larvae (Figure 1C). This indicated that a brief exposure to light can be stressful for larval zebrafish. We replicated these experiments using bPAC^+^ and bPAC^−^ larvae as well as blue-light of increasing light-power. These experiments determined an average 405% (min.: 350%, max.: 439%) and 263% (min.: 230%, max.: 312%) increase of whole-body cortisol level for the bPAC^+^ and bPAC^−^ larvae, respectively. Importantly, yellow-light did not enhance cortisol rise in bPAC^+^ larvae (Figure 3A). Thus, in comparison to their negative siblings, the bPAC^+^ larvae showed a greater cortisol increase in response to blue-light. This happened while the input signal that triggered the rise of cortisol in the first place remained the same for both groups of larvae. Importantly, a 10 times shorter blue-light pulse also caused a light power-dependent enhancement of stress-induced cortisol rise, demonstrating that our protocol can be used to induce fast changes in endogenous GC level (Figure 3B).

Once the stress axis has been activated, GCs feedback onto the brain to limit the release of stress hormones (Dallman and Yates, 1969; Dallman et al., 1994). This feedback is crucial for health, as an excess of GCs is considered a risk factor in humans (Wolkowitz et al., 2009). Studies in humans and other species have shown that prenatal treatment with GCs reduces birth weight and leads to an offspring with altered HPA axis activity and increased risk of cardio-metabolic and psychiatric diseases (Kapoor et al., 2006; Seckl and Holmes, 2007; Seckl, 2008). Moreover, alterations in several brain areas have been reported as a consequence of prenatal stress or injection of synthetic GCs (Cratty et al., 1995; Weaver et al., 2004; Szyf et al., 2005; Kapoor et al., 2006; Murmu et al., 2006). However, since GCs exert pleiotropic developmental effects, it is difficult to distinguish between primary (direct) and secondary effects of GC overexposure. Such a distinction requires suitable model systems and appropriate methods for controlling hypercortisolic states during early development.

Our tests with repeated light stimulation determined that the bPAC^+^ larvae responded to each pulse of blue-light with increased cortisol levels, whereas the bPAC^−^ larvae failed to do so after the first pulse (Figure 4B). These results demonstrated that multiple light stimulations can repeatedly cause hypercortisolic states in bPAC^+^ larvae, even if the HPI axis has already been down-regulated by previously elevated cortisol levels. Moreover, when incubated with the antagonist for the GC-receptor Mifepristone, both groups of larvae responded to each of the several light pulses with increased cortisol, but the bPAC^+^ larvae still showed greater cortisol levels (Figure 4C). These results established that multiple light stimulations cause HPI axis down-regulation and verified that the gain of the stress axis is increased in bPAC^+^ larvae. Our approach thus allows for temporally precise induction of transient hypercortisolaemia, allowing analyses of early GC overexposure on stress response regulation. Strikingly, it can also be used to induce persistent forms hypercortisolaemia in bPAC^+^ larvae if repeatedly applied during earlier stages of the larval development.

GCs are known to mobilize energy (Sapolsky et al., 2000), which is necessary to cope with the high kinetic energy demands frequently associated with stress. Interestingly, our experiments determined that optogenetically elevated cortisol levels led to enhanced locomotion shortly after stressor exposure (Figures 5A–D). Substantial evidence shows that stress and GCs exert significant effects on behavior. But because stressors exert their effects through the closely linked actions of various hormones, not only of GCs, specific GC effects on behavior have been difficult to test. Larval zebrafish offer an excellent opportunity for studying the relationship between stress and behavior, although suitable behavioral endpoints need to be developed. Our protocol can be used alongside novel behavioral tests in order to examine GC effects on stress reactions and coping capacities. It could also be combined with *in vivo* small-molecule behavioral screens (Rihel and Schier, 2012) to find novel modulators of behavioral GC effects.

Optogenetic tools provide hitherto unparalleled means for non-invasive manipulation of neuronal activity. So far, optogenetic applications have been used extensively to modify neuronal activity via light-gated channels. There are comparatively fewer examples of photo-actuators used to manipulate intracellular signaling. Our results demonstrate the feasibility of selectively increasing stress-induced cortisol levels by optogenetic manipulation of cAMP level. The larval zebrafish is highly suitable for non-invasive optogenetics due to its genetic amenability and transparent body (Gahtan and Baier, 2004; Portugues et al., 2013). We showed that the gain of the stress axis can be optogenetically increased in freely behaving larval zebrafish, modifying endocrine and behavioral outputs. So far, bPAC had not been used to modify neuroendocrine and behavioral adjustments in vertebrates. We provide a first demonstration for the feasibility of using it in larval zebrafish to enhance cAMP levels, hormone release and behavioral alteration. Given the availability of a large number of tissue-specific promoters, our protocol could be extended to other cell-types to alter physiological processes *in vivo* using bPAC. Moreover, it could be combined with imaging and bioluminescence techniques for detailed examinations of GC effects on the activity of hypothalamic and pituitary cells.

In summary, our study introduces a powerful tool for the analysis of rapid and delayed GC effects on brain function and behavior, feedbacks within the stress axis and developmental programming by GCs. Follow up work involves analyses of stress circuit development and stress behavior against backgrounds of nominal and increased gain of the HPI axis.

## Author contributions

Conception and design of the experiments: Soojin Ryu and Rodrigo J. De Marco. Acquisition of data: Antonia H. Groneberg, Rodrigo J. De Marco, Chen-Min Yeh, Soojin Ryu, Luis A. Castillo Ramírez. Analysis and interpretation of data: Rodrigo J. De Marco, Soojin Ryu, Antonia H. Groneberg, Chen-Min Yeh, Luis A. Castillo Ramírez. Drafting the article: Rodrigo J. De Marco, Soojin Ryu, and Antonia H. Groneberg.

### Conflict of interest statement

Soojin Ryu and Rodrigo J. De Marco have submitted a patent application for the use of optogenetic transgenic zebrafish lines to find novel regulators of stress response.
